# Supplementary material for: Design of task-specific optical systems using broadband diffractive neural networks
Source: Light Sci Appl. 2019 Dec 2;8:112. doi: 10.1038/s41377-019-0223-1 (PMC6885516; doi:10.1038/s41377-019-0223-1)
Supplement: Supplementary file 1 — Supplementary Information [file 41377_2019_223_MOESM1_ESM.pdf]

Supplementary Information for

Design of Task-Specific Optical Systems Using Broadband Diffractive Neural Networks

Running title: Broadband Diffractive Neural Networks

Yi Luo[1,2,3†] email: yluo2016@ucla.edu

Deniz Mengu[1,2,3†] email: denizmengu@ucla.edu

Nezih T. Yardimci[1,3] email: yardimci@ucla.edu

Yair Rivenson[1,2,3] email: rivensonyair@ucla.edu

Muhammed Veli[1,2,3] email: mveli@ucla.edu

Mona Jarrahi[1,3] email: mjarrahi@ucla.edu

Aydogan Ozcan[1,2,3,4,*] email: ozcan@ucla.edu

telephone: +1 310-825-0915

[1] Electrical and Computer Engineering Department, University of California, Los Angeles, CA, 90095, USA

[2] Bioengineering Department, University of California, Los Angeles, CA, 90095, USA

[3] California NanoSystems Institute, University of California, Los Angeles, CA, 90095, USA

[4] Department of Surgery, David Geffen School of Medicine, University of California, Los Angeles, CA, 90095, USA.

† Equal contributing authors

* Corresponding author

Supplementary Figures

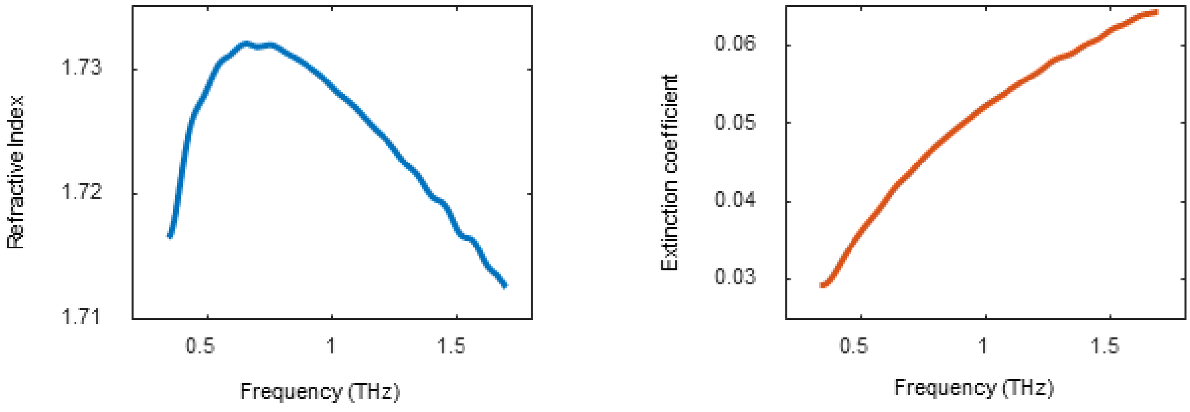

Supplementary Figure S1. Dispersion curves of the polymer material (VeroBlackPlus RGD875) used for 3D-printing of our diffractive optical neural networks. Complex valued refractive index of a 1 mm-thick 3D printed layer that is made of VeroBlackPlus RGD875 was analyzed by the THz-TDS system described in the Methods section of the main text. The refractive index (blue line) and the extinction coefficient (red line) of VeroBlackPlus RGD875 material were extracted from the real and imaginary parts of the complex refractive index, respectively.

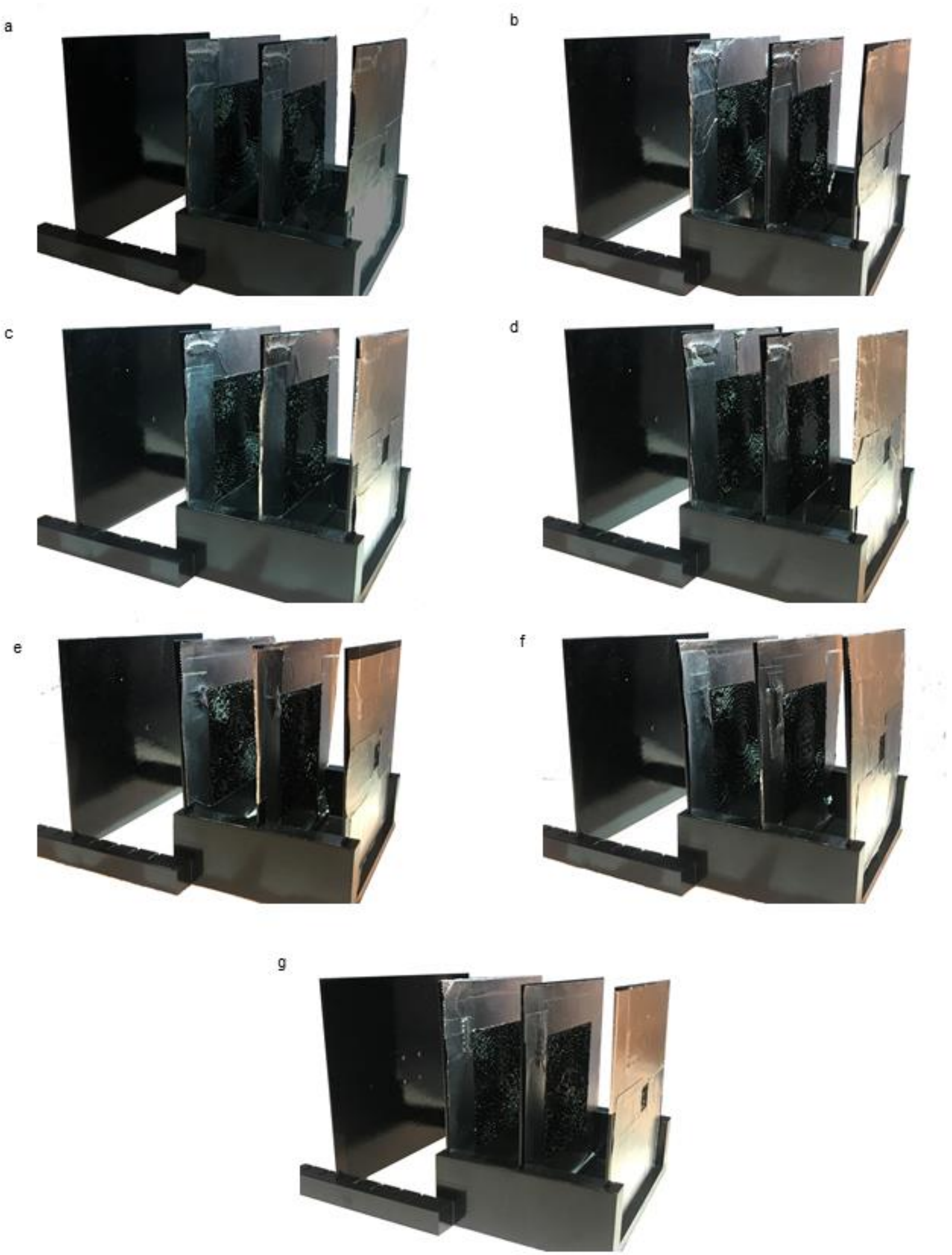

Supplementary Figure S2. Photographs of the fabricated broadband diffractive neural networks. **(a-e)** Single passband spectral filters, with a center frequency of **a** 300 GHz (Figure

2a, main text), **b** 350 GHz (Figure 2b, main text), **c** 400 GHz (Figure 2c, main text), **d** 420 GHz (Figure 2d, main text), **e** 350 GHz (Figure 2e, main text). **(f)** Dual passband spectral filter (Figure 3, main text), and **(g)** spatially-controlled wavelength de-multiplexing system (Figure 4, main text).

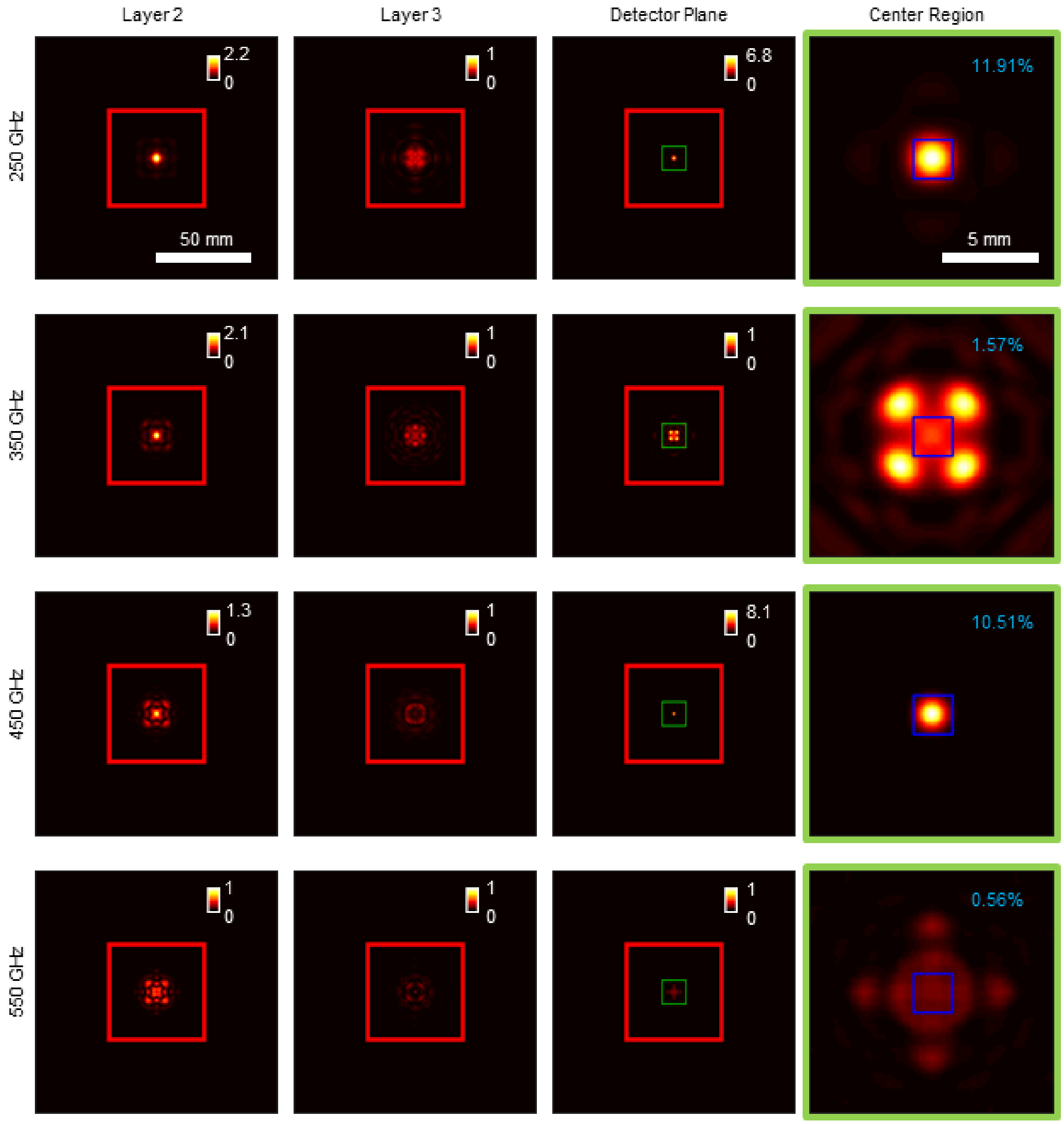

Supplementary Figure S3. Lateral intensity distributions synthesized by a broadband diffractive neural network-based dual passband spectral filter. In this diffractive dual passband filter design, the center frequencies of the target bands are 250 GHz and 450 GHz. From left to right, each column depicts the calculated intensity distributions of the waves

incident at the 2nd and 3rd diffractive layers, the output plane and the 12.5 mm × 12.5 mm region (green squares) around the output aperture (blue squares), respectively, for 4 different frequencies corresponding to the 4 rows, respectively. The total area shown by the images in the first 3 columns corresponds to 128 mm × 128 mm with the central 50 mm × 50 mm portion (red squares) representing the area of the diffractive layers, i.e., the modulation region. The blue squares in the 4th column represent the area of the output aperture (2mm × 2mm) and the optical power that falls within this area with respect to the total input power is reported as a percentage (i.e., the blue percentage values). The x-y plane is sampled at a rate of 0.125 mm on both directions, and the power level within each 0.125 × 0.125 mm^2 area is normalized with respect to the power level contained within an equal area at the corresponding input aperture.

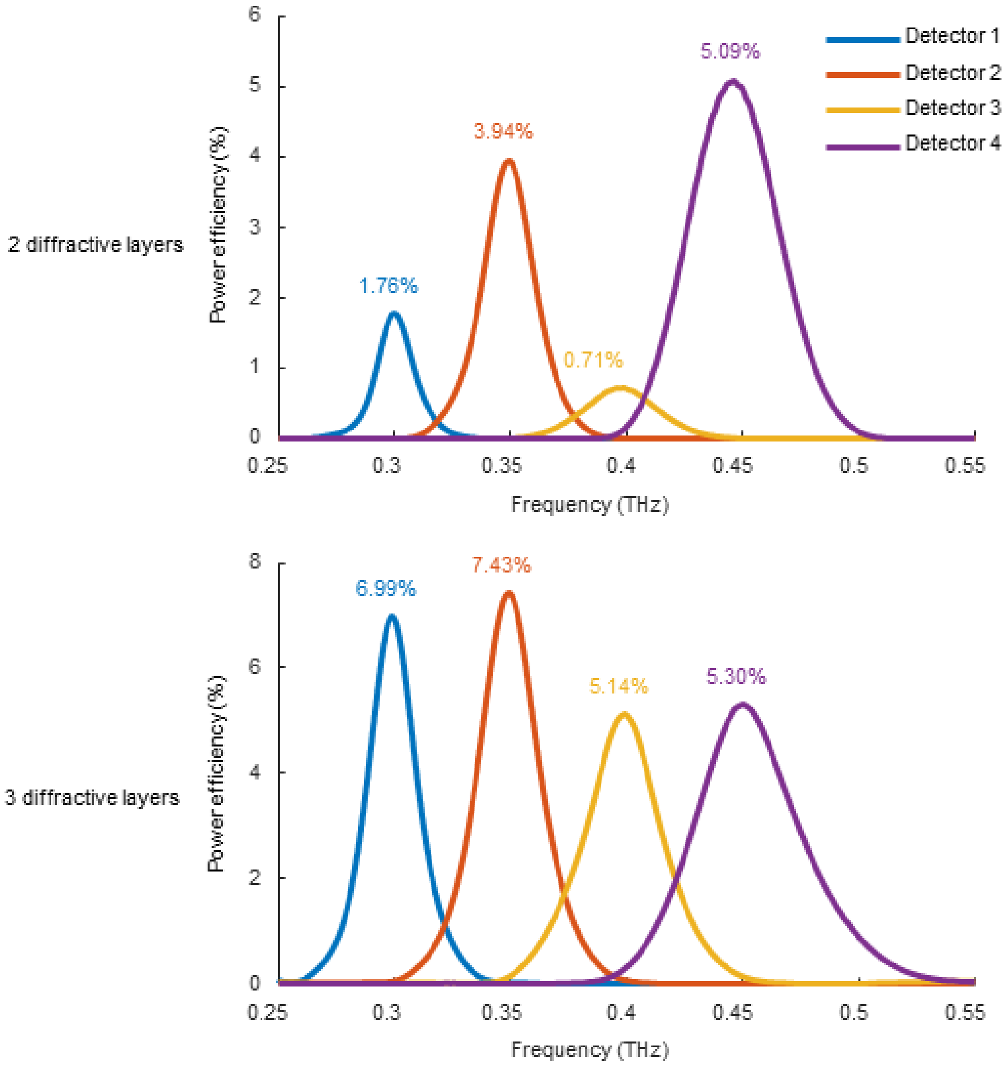

Supplementary Figure S4. Comparison of spatially-controlled wavelength de-multiplexing systems that use two and three diffractive layers. All the other parameters of the system,

including the input/output NA, layer-to-layer spacing, training initialization and the number of epochs etc. remained the same in both cases. The percentage values shown in the figures correspond to the power efficiency of the peak frequency in each band. A two-layer diffractive system is not as successful as the three-layer diffractive system for the same task of controlling the spatial locations of these four de-multiplexed passbands at the output plane of the optical network.

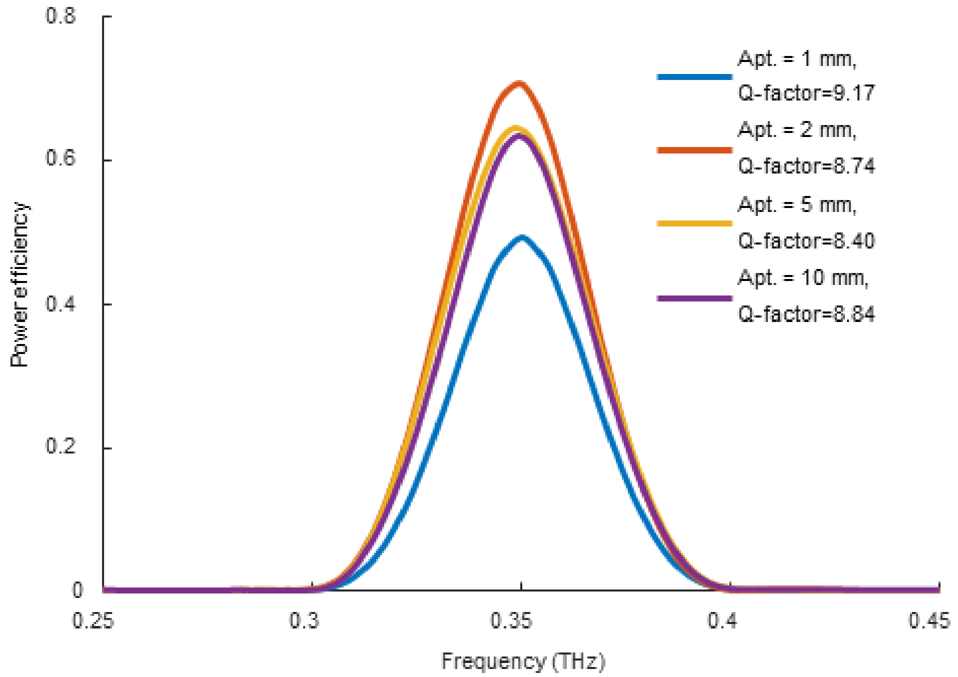

Supplementary Figure S5. Broadband diffractive optical network-based spectral filter design using different sizes of output apertures. Using 3 diffractive layers with a layer-to-layer separation of 3 cm, and an output aperture that is 5 cm away from the 3rd diffractive layer, different single passband spectral filters were designed around the center frequency of 350 GHz by changing the size of the output aperture (1, 2, 5 and 10 mm). Training phase involved a target Q-factor of 10 and $\frac{\alpha}{\beta} = 1$. The degrees of freedom in our diffractive network permit us to maintain the Q-factor for various aperture sizes. Relatively lower power efficiency in the case of the 1 mm aperture (blue curve) is due to the fact that the aperture size in that case is comparable to the center wavelength (~0.85 mm).

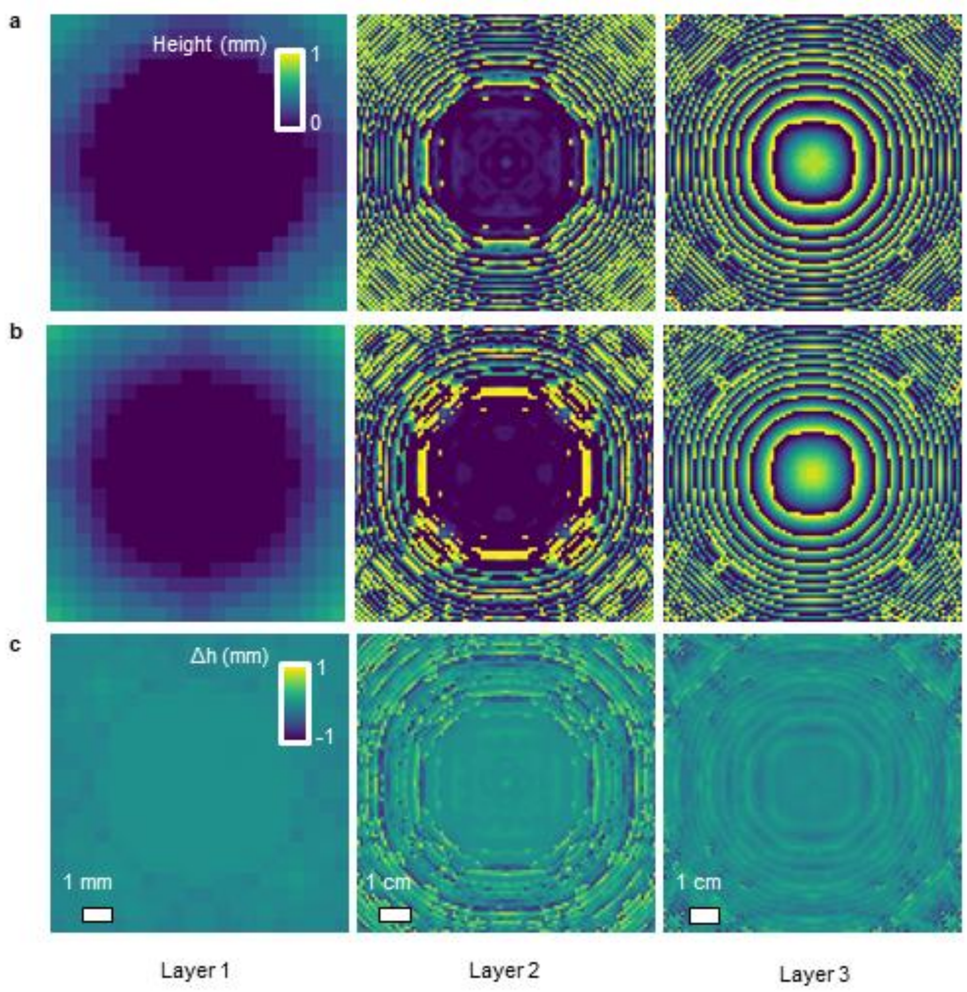

Supplementary Figure S6. Thickness distributions of the diffractive layers of the two single passband filter designs, before and after transfer learning. a Thickness profile of the diffractive layers before transfer learning; also shown in Figure 2b of the main text. **b** Thickness profile of the diffractive layers after transfer learning. **c** The differences in the thickness distributions of the diffractive layers of the two designs, before and after transfer learning. With the additional constraints and training (as detailed in the Discussion section and Figure 5 of the main text), subtle changes to the thickness distributions of the diffractive layers were applied to improve the tunability of the diffractive bandpass filter. The positive (negative) values in (c) denote a thickness reduction (increase) after the transfer learning step, which generated a new design that is specifically engineered for a tunable frequency response with enhanced and a more uniform Q-factor across the targeted axial displacement range (see Figure 5 of the main text).
