# Supplementary material for: Design of task-specific optical systems using broadband diffractive neural networks
Source: Light Sci Appl. 2019 Dec 2;8:112. doi: 10.1038/s41377-019-0223-1 (PMC6885516; doi:10.1038/s41377-019-0223-1)
Supplement: Supplementary file 2 — Research Summary [file 41377_2019_223_MOESM2_ESM.docx]

***Light: Science & Applications***

# Optical computing: Digging deep for object recognition

An optical computing process called Diffractive Deep Neural Networks has been used to develop a machine-learning process based on analyzing light over a wide and continuous range of frequencies, known as broadband light. Aydogan Ozcan and colleagues at the University of California, USA, built their system around a diffractive optical network, which analyses the diffraction of light from a sample as it interacts with several layers of diffracting material. They combined this with a ‘deep learning’ process that extracts and learns from information at increasing levels of complexity. The computation by a neural network mimics the learning processes believed to occur in the brain. The researchers demonstrated the use of the system for several object-recognition tasks. Analyzing light across many frequencies should significantly enhance the ability of the system to recognize complex objects, including handwriting.

**Related article manuscript number:**  LSA20191012R

**Article title:**  Design of Task-Specific Optical Systems Using Broadband Diffractive Neural Networks

**Corresponding author and affiliation/s:** Aydogan Ozcan, University of California Los Angeles, ECE, Los Angeles, United States

**About your Editorial Summary — please read**

**Before approving this Editorial Summary, please carefully check that (1) the summary text lists the correct author(s) and (2) the spelling and order of all author names and affiliations are correct.**

This **Editorial Summary** is based on your manuscript that was recently accepted for publication in *Light: Science & Applications* (LSA). It provides a non-specialist audience with a synopsis of your key research outcomes and conclusions. This value-added service provided by Springer Nature is designed to raise interest in your research across the broader community.

Springer Nature will publish the summary on the journal’s website, and it will be freely available under the CC BY licence (Creative Commons Attribution v4.0 International Licence) (see the journal website for details). We encourage you to re-use the summary to bring attention to your research; for example, you can host it on your own website and share it via social-networking platforms. Please attribute the summary to *LSA* and your article (e.g. by providing a link to your article) and do not make derivatives.

Please note that to maximise the usefulness of these summaries they must follow several stringent guidelines:
-- Spelling, punctuation and style are set according to *Nature* editorial guidelines. As this summary is aimed at non-expert readers, some concepts and technical terms will be simplified.
-- Total length must be no more than 135 words. It is likely that not all points in the paper will be covered.
-- The first sentence must be no more than 280 characters, including spaces, to allow use on microblogging sites.
-- The headline must consist of a brief generic subject identifier followed by a short description. No more than 10 words in total.

Please contact the editorial office () immediately with corrections should you find any factual errors in this Editorial Summary.
